# Genetic Co-Administration of Soluble PD-1 Ectodomains Modifies Immune Responses against Influenza A Virus Induced by DNA Vaccination

**DOI:** 10.3390/vaccines8040570

**Published:** 2020-10-01

**Authors:** Pierre Tannig, Antonia Sophia Peter, Dennis Lapuente, Stephan Klessing, Anna Schmidt, Dominik Damm, Matthias Tenbusch, Klaus Überla, Vladimir Temchura

**Affiliations:** Institute of Clinical and Molecular Virology, Friedrich-Alexander-University Erlangen-Nürnberg, 91054 Erlangen, Germany; pierre.tannig@fau.de (P.T.); antoniasophia.peter@uk-erlangen.de (A.S.P.); dennis.lapuente@uk-erlangen.de (D.L.); stephan.klessing@uk-erlangen.de (S.K.); anna.schmidt@extern.uk-erlangen.de (A.S.); dominik.damm@uk-erlangen.de (D.D.); matthias.tenbusch@fau.de (M.T.); klaus.ueberla@fau.de (K.Ü.)

**Keywords:** influenza A, DNA vaccine, DNA adjuvants, checkpoint blockade, soluble PD-1, soluble PD-L1, intramuscular electroporation, immunomodulation

## Abstract

Due to the low efficacy and the need for seasonal adaptation of currently licensed influenza A vaccines, the importance of alternative vaccination strategies is increasingly recognized. Considering that DNA vaccines can be rapidly manufactured and readily adapted with novel antigen sequences, genetic vaccination is a promising immunization platform. However, the applicability of different genetic adjuvants to this approach still represents a complex challenge. Immune checkpoints are a class of molecules involved in adaptive immune responses and germinal center reactions. In this study, we immunized mice by intramuscular electroporation with a DNA-vaccine encoding hemagglutinin (HA) and nucleoprotein (NP) of the influenza A virus. The DNA-vaccine was applied either alone or in combination with genetic adjuvants encoding the soluble ectodomains of programmed cell death protein-1 (sPD-1) or its ligand (sPD-L1). Co-administration of genetic checkpoint adjuvants did not significantly alter immune responses against NP. In contrast, sPD-1 co-electroporation elevated HA-specific CD4^+^ T cell responses, decreased regulatory CD4^+^ T cell pools, and modulated the IgG2a-biased HA antibody pattern towards an isotype-balanced IgG response with a trend to higher influenza neutralization in vitro. Taken together, our data demonstrate that a genetic DNA-adjuvant encoding soluble ectodomains of sPD-1 was able to modulate immune responses induced by a co-administered influenza DNA vaccine.

## 1. Introduction

Outbreaks of viruses with pandemic potential are a persistent burden for global health systems [1,2,3,4,5]. The development of prophylactic vaccines against those viruses is often time-consuming and, in the case of influenza A viruses (IAV), complicated by continuous alterations of surface proteins via antigenic shift and drift [6,7]. In this regard, genetic vaccination offers a way to accelerate vaccine development [8]. However, there has been limited success in the clinical development of DNA vaccines for influenza. Contributing factors appear to include an unfavorable antibody subtype pattern induced by viral surface protein sequences used in a vaccine composition [9,10] and the relatively weak immunogenicity of DNA-vaccines in humans [11]. Although application methods like gene gun and electroporation have already significantly improved DNA vaccination efficacy, genetic adjuvants might offer an additional way to increase and influence the quality and amplitude of immune responses [12,13,14,15,16]. However, the adjuvant-mediated modulation of influenza DNA vaccine responses still poses a challenging task [17].

Application of checkpoint inhibitors (CPIs) that are broadly used for the treatment of various cancers [18,19] due to their ability to restore T cell functions in the tumor microenvironment, might be prospective candidates for the modulation of vaccine-induced immune responses. Recently, we demonstrated that the interference with these inhibitory interactions might be a platform to enhance HIV-1-specific immune response. By blocking of PD-1 and its ligands via co-application of plasmids encoding for the soluble ectodomains of PD-1 (sPD-1) or PD-L1 (sPD-L1) during HIV-1 DNA-immunizations, we demonstrated that sPD-L1 modulated HIV Env-specific T-cell and antibody responses [20]. However, HIV-1 Env resembles an atypical viral surface antigen given its vast sequence diversity and extensive glycosylation profile structurally distinguishing it from glycoproteins of respiratory viruses [21]. Moreover, there are also substantial differences in regard to vaccine-induced immune responses with Env immunizations resulting in a T_H_2-biased adaptive immune response compared to the T_H_1-bias observed after immunizations against hemagglutinin of the influenza A virus (HA) and F-protein of the respiratory syncytial virus (RSV-F) [21,22,23].

In this study, we applied soluble PD-1 and PD-L1 ectodomains as DNA-encoded adjuvants for genetic vaccination against the hemagglutinin and nucleoprotein (NP) of IAV. This resulted in an enhancement of HA-specific T and B cell responses and shifted the T_H_1-biased HA-specific antibody subtype pattern towards a more balanced antibody response.

## 2. Materials and Methods

### 2.1. Mice Housing, Immunizations and Ethics Statement

Five to six-week old BALB/c mice from Charles River Laboratories (Wilmington, NC, USA), were used in this study and housed in accordance with the national law and institutional guidelines at the Franz-Penzoldt-Center of the Faculty of Medicine, University Clinics Erlangen (Erlangen, Germany) and at the animal facility of the Faculty of Medicine, Ruhr University Bochum (Bochum, Germany).

DNA immunizations were performed as described previously [20]. Briefly, mice were anesthetized and electroporated with the TriGrid electrode array (Ichor Medical, San Diego, CA, USA). 30–45 µg of plasmid DNA in a total volume of 60 µL were injected intramuscularly followed by the immediate application of electrical impulses at the administration site.

All conducted animal experiments were approved by the Government of Lower Franconia according to the license 55.2-2532-2-203 and by an external ethics committee authorized by the North Rhine-Westphalia State Office for Consumer Protection and Food Safety (license 84-02.04.2013-A371).

### 2.2. Plasmids

For DNA immunization, codon-optimized expression vectors pVax-HA encoding hemagglutinin (pHA) and pVax-NP encoding nucleoprotein (pNP) of the influenza A virus (strain A/Puerto Rico/8/1934/H1N1) were used together with expression plasmids for the soluble ectodomains of PD-1 or PD-L1 [20]. As a mock control, the pVax vector system with an empty expression cassette was used. In vivo tracing HA and NP plasmids pDP-LUC-HA and pDP-LUC-NP additionally encoding luciferase were used to monitor influenza antigen expression in vivo. As a control, the luciferase-encoding plasmid pDP-LUC-empty was used [20].

By enzymatic restriction of a pVax plasmid encoding HA of the influenza A virus (strain A/Puerto Rico/8/1934/H1N1) (UniProtKB/Swiss-Prot Sequence ID: P03452.2) using XbaI and EcoRI (both New England Biolabs, Ipswich, MA, USA), an HA fragment without transmembrane domain (TM) (deletion of QILAIYSTVASSLVLLVSLGAISFWMCSNGSLQCRICI at the C-terminus) was obtained. This fragment was cloned into the pVax vector system containing a Kozak sequence and tissue plasminogen activator (TPA) leader motif in order to increase antigen secretion (Supplementary Figure S2A).

### 2.3. Cell Culture

Human embryonic kidney cells (HEK 293T, obtained from European Collection of Cell Cultures, Salisbury, UK) and MDCK-II cells (ATCC^®^ CRL-2963™) were cultured in DMEM (Gibco, ThermoFisher Scientific, Waltham, MA, USA) supplemented with 10% FCS (Sigma Aldrich, Taufkirchen, Germany), 1% penicillin/streptomycin (Sigma Aldrich, Taufkirchen, Germany), and 2 mM L-glutamine (Gibco, ThermoFisher Scientific, Waltham, MA, USA)).

FreeStyle 293F (obtained from Thermo Fisher, Schwerte, Germany) cells were cultured as recommended by the manufacturer protocol mildly stirring in a humidified 8% CO_2_ atmosphere. The cells were maintained in a density between 0.5 and 2 × 10^6^ cells/mL.

### 2.4. Analysis of Antigen Expression In Vivo

Influenza antigen expression was analyzed as described before [17,20]. Briefly, mice received 20 µg of luciferase-encoding plasmid by intramuscular electroporation. Subsequently, 200 µg D-luciferin was injected into the hind legs at the indicated time points after immunization. After 3 min, luminescence signals representing a proxy of influenza antigen expression were assessed and quantified using an IVIS Lumina Series II (PerkinElmer, Waltham, MA, USA).

### 2.5. Protein Production and Purification

FreeStyle 293F cells were transfected with 80 µg of expression plasmids encoding for soluble HA without transmembrane domain (HA-TM) in sterile disposable PETG flasks (Wagner and Munz GmbH, Munich, Germany) with 3 µg polyethylenimine (Sigma Aldrich, Taufkirchen, Germany) per 1 µg DNA in OPTI-MEM Reduced Medium (Thermo Fisher, Schwerte, Germany). Culture medium was exchanged six hours after transfection. Three days post-transfection, supernatants were collected and sterile filtered through 0.2-µm Minisart filters (Sigma Aldrich, Taufkirchen, Germany) before purification via Erythrina cristagalli (Vector Laboratories Inc., Burlingame, CA, USA) lectin affinity chromatography. After washing with PBS containing 1 mM EDTA and 1 mM EGTA (Sigma Aldrich, Taufkirchen, Germany), columns were loaded with the filtered supernatant. Columns were washed and protein eluted using 200 mM lactose (Sigma Aldrich, Taufkirchen, Germany). Protein samples were concentrated and elution carbohydrates in the eluate dialyzed via Amicon Centrifugal Filters with 10 kDa cut-off (Merck, Darmstadt, Germany). Protein concentration was analyzed using the ND100-NanoDrop^®^ (peQlab, Erlangen, Germany). Samples were stored at 4 °C until further use. Protein production was monitored with Western Blot and purity assessed by Silver staining (Supplementary Figure S1C).

### 2.6. ELISA-Based Antibody Assay

At the indicated time-points, mice were bled by puncturing of the retro orbital sinus using heparinized capillaries (Hirschmann Laborgeräte, Eberstadt, Germany). Blood samples were centrifuged for 5 min at 2370× *g* and sera were stored at −20 °C until further use. The antigen-specific antibody production was measured by a HA and NP ELISA using purified soluble HA or recombinant His-tagged NP (Sino Biological Inc., Peking, China) as a coating antigen. Quantitative analysis of the HA-specific IgG1 amounts was performed using a monoclonal anti-influenza Hemagglutinin antibody (2F1A7, IgG1, Sino Biological, Peking, China). High-binding 96-well microtest plates (Sarstedt, Nümbrecht, Germany) were coated with 100 ng of HA or NP in bicarbonate buffer (pH 9.6) at room temperature overnight. After washing with PBS containing 0.05% Tween20 (PBS-T), blocking with 5% skimmed milk was performed. After washing, serum samples were diluted in 2% skimmed milk and incubated. The HRP-conjugated secondary antibodies directed against IgG1, IgG2a, IgG2b, and IgG3 (Southern Biotech, Birmingham, AL, USA) were used in equal amounts to detect the respective antibody subtypes. Finally, after the plates were washed, relative light units (RLUs) were measured with the multilabel plate reader Victor (Perkin Elmer, Hamburg, Germany).

### 2.7. Analysis of Cellular Immune Responses

Intracellular cytokine staining (ICS) was used to detect influenza HA-specific T cell responses in the spleens. After mice were sacrificed, splenic single-cell suspensions were prepared by homogenization through a 70 µm cell strainer (Corning Inc., Corning, Harrodsburg, KY, USA). After erythrocyte lysis, splenocytes were resuspended in RPMI 1640 (Gibco, ThermoFisher Scientific, Waltham, MA, USA) supplemented with 10% FCS (Sigma Aldrich, Taufkirchen, Germany), 1% penicillin/streptomycin (Sigma Aldrich, Taufkirchen, Germany), 10 mmol HEPES (Gibco, ThermoFisher Scientific, Waltham, MA, USA), 2 mmol L-glutamine (Gibco, ThermoFisher Scientific, Waltham, MA, USA), and 50 µmol β-Mercaptoethanol (PAN-Biotech, Aidenbach, Germany).

In a 96-well U-bottom microtiter plate (Greiner Bio-One, Frickenhausen, Germany), 10^6^ splenocytes/well were seeded and stimulated with 5 μg/mL of the MHC-II restricted influenza peptides HA_110–120_ (SFERFEIFPKE) and NP_55–69_ (RLIQNSLTIERMVL) or MHC-I restricted influenza peptides HA_518–526_ (IYSTVASSL) and NP_147–155_ (TYQRTRALV) in the presence of 2 μg/mL anti-CD28 (37.51; eBioscience, San Diego, CA, USA) and 3 μg/mL Brefeldin A (eBioscience, San Diego, CA, USA) for 6 h at 37 °C in a humidified 5% CO_2_ atmosphere. After stimulation, staining with anti-mouse CD4 BV650 (RM4-5, Biolegend, San Diego, CA, USA) and Fixable Viability Dye eFluor 450 (eBioscience, San Diego, CA, USA) was performed. Cells were fixed with 2% paraformaldehyde and permeabilized with 0.5% saponin (Sigma Aldrich, Taufkirchen, Germany) in the presence of 1.7 µg/mL anti-mouse CD16/CD32 (93, eBioscience, San Diego, CA, USA). For ICS, cells were stained with anti-mouse TNFα PE-Cy7 (MP6-XT22), anti-mouse IL-2 APC (JES6-5H4), and anti-mouse IFNγ PE (XMG1.2, all from eBioscience, San Diego, CA, USA) in 0.5% saponin. Cytokine production was assessed by FACS-LSR II (BD, Franklin Lakes, NJ, USA) and data was analyzed using FlowJo (Tree Star, Ashland, OR, USA).

### 2.8. Microneutralization Assay

The influenza A microneutralization assay was performed as described previously [24]. Briefly, 5 × 10^4^ MDCK-II were seeded in 96-well F-bottom plates (Sarstedt, Nümbrecht). On the next day, a 2-fold serial dilution of serum samples was prepared and incubated for 45 min at 37 °C with 2 × 10^3^ PFU/well influenza A/PuertoRico/8/34 before adding the mixture to the cells. After 1.5 h, DMEM containing 0.18% BSA, 1% penicillin/streptomycin and 1.2 μg/mL Trypsin (Gibco, ThermoFisher Scientific, Waltham, MA, USA) was changed and cells incubated for 4 days. CPE was assessed by crystal violet staining and the reciprocal serum dilution completely inhibiting infection considered as the neutralizing antibody titer.

### 2.9. Staining of Regulatory T Cells and Memory B cells

Regulatory T cell staining was performed as previously described [20]. Briefly, 10^6^ splenocytes/well were seeded in 96-well U-bottom plates and stained with anti-mouse CD4 BV650 (RM4-5, Biolegend, San Diego, CA, USA), anti-mouse CD25 APC (PC61, BD Pharmingen, San Jose, CA, USA), and Fixable Viability Dye eFluor450 (eBioscience, San Diego, CA, USA). Cells were fixed, permeabilized and stained intracellularly with anti-mouse Foxp3 PE (MF14, Biolegend, San Diego, CA, USA). To measure samples, FACS-LSR II (BD, Franklin Lakes, NJ, USA) was used and data analyzed using FlowJo (Tree Star, Ashland, OR, USA).

For memory B cell staining, 10^6^ splenocytes/well were seeded in 96-well U-bottom plates and surface stained with anti-mouse CD19 Qdot655 (6D5, Thermo Fisher Scientific, Schwerte, Germany), anti-mouse CD80 APC (16-10A1, BD, Franklin Lakes, NJ, USA), anti-mouse IgD PE (217–170, BD, Franklin Lakes, NJ, USA) and Fixable Viability Dye eFluor450 (eBioscience, San Diego, CA, USA). HA-specific B cells were detected by surface staining with soluble HA of the influenza A virus (strain A/Puerto Rico/8/1934/H1N1) labeled with Alexa488 using the Alexa488 Protein Labeling Kit (Thermo Fisher, Schwerte, Germany).

### 2.10. Statistical Analysis

Data are presented as means ± standard errors of means (SEM). As indicated in the figure legends, statistical analysis was performed with GraphPad Prism software version 7 (Graphpad Software Inc., San Diego, CA, USA) using one-way analysis of variance (ANOVA) with Tukey’s post-test or unpaired *t* tests.

## 3. Results

### 3.1. HA-Specific T Cell Responses Are Enhanced After sPD-1 Co-Electroporation

**Figure 1:**
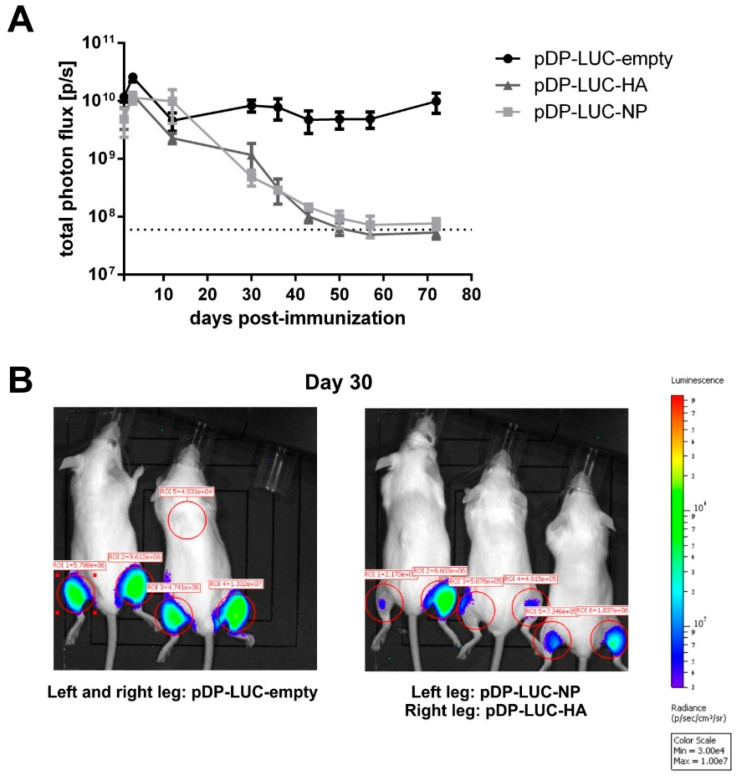
DNA electroporation of influenza antigens: in vivo antigen expression and immunogenicity. (**A**) Long-term antigen expression after DNA electroporation. BALB/c mice (*n* = 2–3) were intramuscularly electroporated with 20 µg of the luciferase-encoding plasmids pDP-LUC-empty, pDP-LUC-HA or pDP-LUC-NP. Luminescence signals were quantified in the hind legs of immunized mice at indicated time-points after electroporation. The dotted line represents background luminescence. (**B**) Two BALB/c mice were intramuscularly electroporated in both hind legs with 20 µg luciferase-encoding plasmid pDP-LUC-empty, three BALB/c mice received an intramuscular electroporation of 20 µg pDP-LUC-NP in the left hind leg and 20 µg pDP-LUC-HA in the right hind leg. Luminescence signals were quantified in the red-circled areas, here shown 30 days after electroporation. Background luminescence is shown in ROI = 5.

For genetic immunization against influenza A virus, we selected a DNA vaccine consisting of HA and NP expressing plasmids (pHA and pNP) [17]. First, we monitored the durability of in vivo expression for influenza antigens after DNA electroporation. To trace the presence of the antigens in muscle tissues, luciferase-encoding pDP-LUC-HA and pDP-LUC-NP plasmids were used. The control luciferase encoding plasmid pDP-LUC-empty demonstrated a durable rate of expression over 70 days, whereas we observed a continuous decline of luciferase signal intensity for HA and NP encoding plasmids (Figure 1A,B). This decline could be potentially caused by CTL-mediated killing of antigen-producing cells (Supplementary Figure S1).

**Figure 2:**
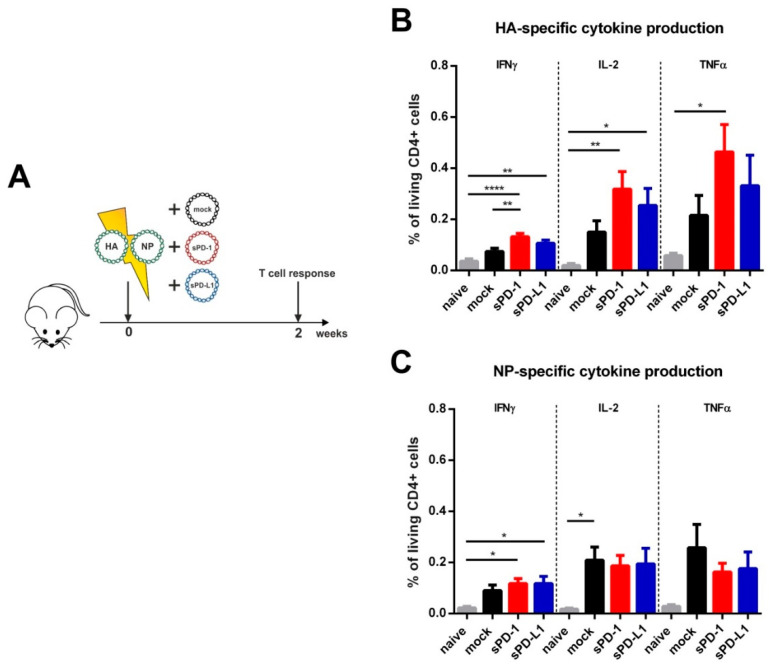
Immunization outline and HA- and NP-specific CD4 T cell responses. (**A**) Six-week old BALB/c mice were electroporated intramuscularly with expression plasmids encoding for HA and NP. Additionally, the animals were either co-electroporated with an empty vector (mock) or plasmids encoding for the soluble ectodomains of PD-1 (sPD-1) or PD-L1 (sPD-L1). After two weeks, mice were sacrificed, and T cell responses analyzed. Percentage of CD4 + T cells producing IFNγ, IL-2 or TNFα after in vitro stimulation with influenza HA (**B**) and NP (**C**) T helper peptides (measured by intracellular cytokine staining). Shown are mean values with SEM (*n* = 5–9) and significant differences between the groups (one-way ANOVA analyses followed by Tukey’s multiple comparison test, * p < 0.05, ** p < 0.01, **** p < 0.0001).

Previously, we reported CPI-mediated effects on antigen-specific T cell responses early after HIV-1 DNA-immunization [20]. In order to analyze the effect of soluble checkpoint ectodomains on influenza-specific T cell responses, mice were immunized by intramuscular electroporation with the influenza DNA vaccine together with sPD-1 or sPD-L1 as genetic adjuvants. To control plasmid-driven effects, the empty pVax vector system (mock) was co-transfected with the influenza DNA vaccine. T cell responses were analyzed two weeks after immunization (Figure 2A). After re-stimulation with an MHC class II restricted immunodominant HA peptide, we detected a significantly higher frequency of splenic CD4 T cells secreting IFNγ compared to mock-adjuvanted animals (Figure 2B). Also, the frequency of IL-2 and TNFα producing cells were elevated in the sPD-1 group. sPD-L1 as a genetic adjuvant also slightly (non-significantly) increased HA-specific CD4 T cell cytokine responses (Figure 2B). For NP-specific CD4 T cell responses however, no significant differences between the group immunized with the mock adjuvant immunized and the CPI adjuvanted groups were detectable (Figure 2C). Although the influenza DNA vaccine induced detectable HA/NP-specific CD8 T cell responses, they were not modulated by soluble immune checkpoint plasmid co-electroporation (Supplementary Figure S1). These data indicate that the modulatory capacity of soluble PD-1 ectodomains was rather engaged at the level of early HA CD4 T cell responses.

### 3.2. Soluble Checkpoint Molecules As Genetic Adjuvants Affect HA- But Not NP-Specific Antibody Responses

**Figure 3:**
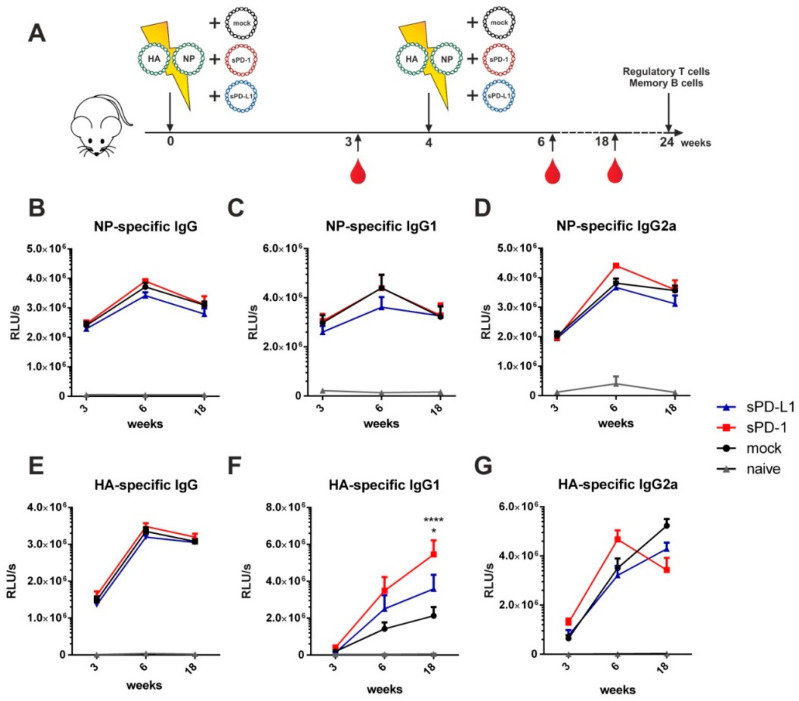
Soluble checkpoint co-expression modulates HA-specific antibody responses. (**A**) Six-week old BALB/c mice were electroporated intramuscularly with expression plasmids encoding for HA and NP together either with an empty vector (mock) or plasmids encoding for sPD-1 or sPD-L1. Four weeks after priming, a booster immunization was administered Blood was drawn at weeks 3, 6 and 18 and antibody responses were analyzed by ELISA. NP-specific IgG (**B**), IgG1 (**C**) and IgG2a (**D**) antibody responses and HA-specific IgG (**E**), IgG1 (**F**) and IgG2a (**G**) antibody responses in the sera of BALB/c mice after i.m. electroporation over a time-period of 18 weeks. Shown are mean values with SEM (*n* = 9–18) and significant differences between immunized groups (two-way ANOVA analyses followed by Tukey’s multiple comparison test, (**F**) * *p* < 0.05 for sPD-L1 group compared to sPD-1 group, **** *p* < 0.0001 for sPD-1 group compared to mock).

In order to evaluate the effects of immune checkpoint modulators on humoral immune responses, we conducted a prime-booster immunization regimen by co-electroporation of the influenza DNA-vaccine together with the genetic checkpoint adjuvants at weeks 0 and 4, respectively (Figure 3A). For analysis of IgG serum antibodies directed against properly glycosylated HA protein, we produced a coating antigen in a eukaryotic cell line. The coating antigen was obtained by cloning of the pHA sequence without its transmembrane domain (TM) into the pVax vector system containing a Kozak sequence and TPA leader motif to increase antigen secretion (Supplementary Figure S2A). This newly generated construct was first transfected into 293T cells. In contrast to HA with TM, the expression of HA was mostly shifted to the supernatant indicating an efficient secretion (Supplementary Figure S2B). In order to produce sufficient amounts of coating antigen, HA-TM was produced in 293F cells and purified over lectin affinity chromatography. Protein abundance and purity was validated by Western blot and silver staining (Supplementary Figure S2C). His-tagged NP coating antigen was purchased commercially.

**Figure 4:**
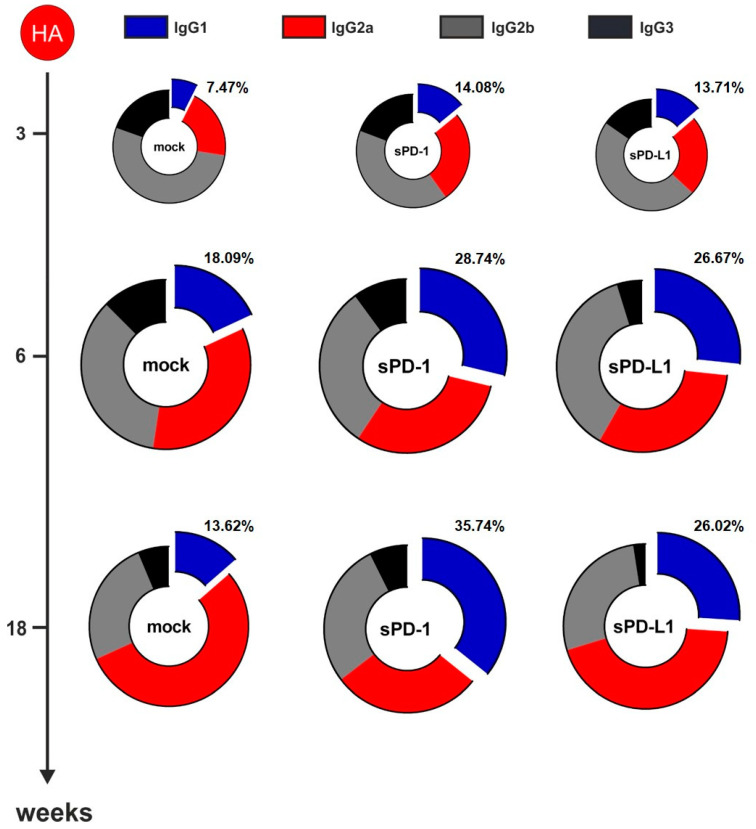
PD-1 co-expression results in a more balanced HA-specific antibody response. Antibody subtype patterns of HA-immunized mice three weeks after priming and two (week 6) and 14 weeks (week 18) after the booster immunization. The ring size represents the overall antibody response. Shown are mean percentages of 18 animals from three independent experiments. Each subtype was analyzed by ELISA with identical amounts of HRP-conjugated anti-mouse IgG1 (blue), IgG2a (red), IgG2b (gray), and IgG3 (black) antibodies.

For NP-specific total IgG (Figure 3B) as well as for IgG1 (Figure 3C), IgG2a (Figure 3D), IgG2b (Supplementary Figure S4A), and IgG3 (Supplementary Figure S4B) antibody subclasses, no significant difference between animals co-electroporated with the genetic checkpoint adjuvants and the mock adjuvant was observed. Despite the HA-specific total IgG not being affected by soluble checkpoint co-expression (Figure 3E) we observed changes in the antibody subtypes elicited by immunization. Co-administration of sPD-1 resulted in a significant increase of HA-specific IgG1 antibody levels (Figure 3F). Significant increase of HA specific IgG1 antibody amounts after sPD-1 co-administration was also confirmed quantitatively (Supplementary Figure S3). Simultaneously, the HA-specific IgG2a responses were decreased in the checkpoint-co-electroporated groups (Figure 4G). HA-specific IgG2b and IgG3 antibody levels were also enhanced in sPD-1 co-electroporated animals (Supplementary Figure S4C,D). The overall antibody subtype pattern of the HA-specific IgG antibodies monitored during the prime-booster immunization regimen resulted in a shift to a more balanced antibody response after soluble checkpoint co-electroporation (Figure 4).

Taken together, administration of the influenza DNA-vaccine together with genetic checkpoint adjuvants significantly affects HA- but not NP-specific vaccine-mediated antibody responses.

### 3.3. sPD-1 Co-Electroporation Enhances Neutralization Titers In Vitro

**Figure 5:**
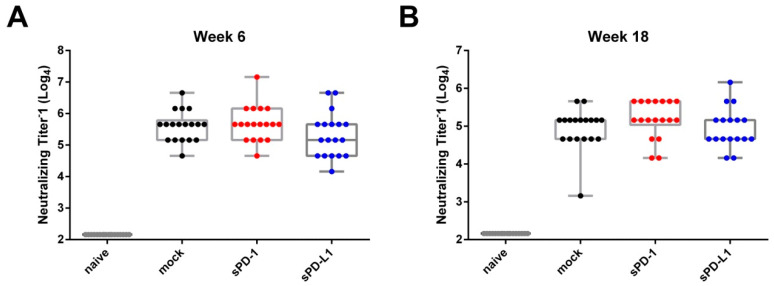
Antibody-mediated neutralization of influenza. Homologous neutralizing titers of vaccine-induced antibodies against influenza A/PR/8/34 shown at week 6 (**A**) and week 18 (**B**). Each dot represents an individual mouse. Shown are whisker plots with median and minimum to maximum distribution (*n* = 17–18).

To analyze the neutralizing capacity of the induced antibodies, we performed a microneutralization assay of the obtained sera [25]. Here, we observed in all immunization groups the highest neutralization titers two weeks after boosting (Figure 5A). sPD-1 co-electroporation resulted in a non-significant trend towards higher neutralization titers compared to mock-treated animals observable at later time points after vaccination (Figure 5B). The number of animals capable of neutralizing influenza at higher reciprocal titers is overall increased in the sPD-1 serum samples (2 animals with neutralizing titer of 2400 in mock group, seven animals in sPD-1 group) (Supplementary Table S1). This trend towards an enhanced neutralization capacity of sPD-1 serum samples might be attributed to the quality of the elicited antibody response.

### 3.4. Effect of sPD-1 Co-Electroporation on Regulatory T Cells and Memory B Cells

**Figure 6:**
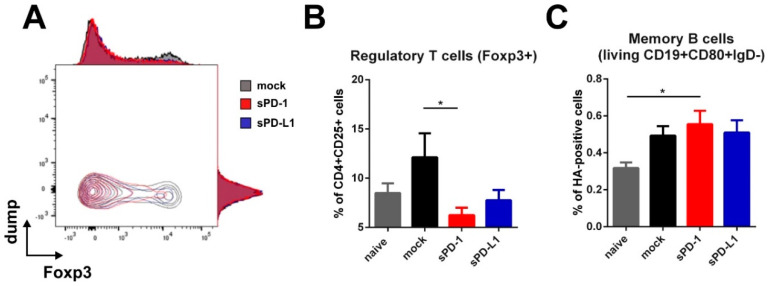
Regulatory T cells and memory B cells are modulated by soluble PD-1. Foxp3 expression among CD4 + CD25 + T cells shown as overlaying blots and histograms (**A**) and percentage of Foxp3-expressing regulatory T cells (within living CD4 + CD25 + cells) (**B**) in the spleen of BALB/c mice 20 weeks after boosting. Shown are mean values with SEM (*n* = 18) and significant differences between groups (one-way ANOVA analyses followed by Tukey’s multiple comparison test, * *p* < 0.05). (**C**) Frequency of HA-specific memory B cells (living CD19 + CD80 + IgD-HA+) in the spleens of BALB/c mice 20 weeks after boosting. Shown are mean values with SEM (*n* = 12) and significant differences between groups (one-way ANOVA analyses followed by Tukey’s multiple comparison test, * *p* < 0.05).

Since the HA-specific antibody subtype patterns were affected up to 14 weeks after boosting, our interest was drawn towards persisting immune cells elicited at later time-points after immunization. For that, we analyzed regulatory T cells and HA-specific memory B cells in the spleens of immunized mice 20 weeks after boosting. Here we observed a significant decrease of regulatory T cells in animals which received the sPD-1 DNA-adjuvant (Figure 6A,B). In mice treated with sPD-L1 DNA, this effect was also present, although it did not reach statistical significance compared to the mock-treated group (Figure 6B). Simultaneously, there was a non-significant trend towards enhanced HA-specific memory B cell frequencies in animals receiving PD-1 DNA co-application (Figure 6C).

## 4. Discussion

With up to 650,000 influenza-associated deaths annually, influenza A still poses a substantial burden for global health systems [26]. Given the high antigenic variability of the surface glycoproteins, HA, strain-specific seasonal vaccines do not ensure protection against heterologous IAV infections [7,27,28,29]. The low vaccine efficiency together with a lengthy and resource-intensive manufacturing process furthermore confirms the need for alternative vaccination platforms resulting in broad protection [30,31,32,33,34].

Genetic vaccination against influenza A might be an advantageous approach compared to the protein-based vaccines manufactured in a cell- or egg-based production process. It represents a cost-effective, time-saving and highly modifiable delivery platform and ensures durable antigen and adjuvant expression in vivo [35]. Due to the capacity to induce protective humoral and cellular immune responses, genetic vaccination is a new frontier in human and veterinary vaccine technology [36,37,38]. In addition, the first DNA vaccine against H5N1 IAV for chickens has been conditionally approved by the USDA recently [39].

Classical adjuvants of protein-based vaccines (like aluminum salts) are poorly applicable for DNA vaccines due to fundamental differences in the antigen delivery mode [38]. Co-administration of plasmids encoding immunomodulatory molecules is a well-accepted way to further improve and modulate DNA-vaccine induced immune responses [40]. However, genetic adjuvantation remains a challenging task.

Checkpoint inhibitors constitute an immunomodulatory platform for the treatment of melanoma and other cancers [18,19]. These are mainly used as monoclonal antibodies to target immune checkpoints expressed on the surface of cancer and immune cells in the tumor microenvironment [41]. The surface expression of immune checkpoints is also enhanced during viral infections, resulting in T cell exhaustion and reduced antiviral responses [42,43,44]. It has been shown by McNally et al. that primary airway epithelial cells strongly express PD-L1 upon influenza infection and blocking this inhibitory ligand by an anti-PD-L1 antibody resulted in enhanced T cell responses and viral clearance [45]. For our DNA vaccination, we adapted a genetic checkpoint inhibitor approach based on the co-electroporation of DNA encoding the soluble PD-1 (sPD-1) and PD-L1 (sPD-L1) ectodomains [20]. The expression of soluble immune checkpoints has already been utilized to block immune checkpoint interactions in vivo and in vitro [46,47,48]. Using this strategy, we previously showed that upon sPD-L1 co-electroporationHIV-1 Env-specific T cell responses were enhanced and antibody responses shifted from a T_H_2-bias towards a more balanced subtype pattern [20].

In this study, we immunized mice by intramuscular electroporation of DNA encoding for influenza HA and NP and co-administered sPD-1 and sPD-L1 plasmids. Opposed to HIV-1 Env, T cell and antibody responses against HA were predominantly modulated by sPD-1. One reason for the observed variations in modulatory activities between soluble PD-1 and PD-L1 adjuvants could stem from the form of the encoded antigens. The application of DNA vaccines against HIV-1 may lead to production and secretion of HIV VLPs in the muscle tissues in situ [20,49,50]. In vitro, co-transfection of 293T cells with HIV-1 Env and Gag plasmids used for the HIV-1 DNA vaccine led to VLP production [20]. In contrast, co-transfection with pHA and pNP plasmids (the influenza A DNA vaccine) did not result in a detectable particle production (data not shown).

Another explanation might be the intrinsic property of HIV Env to modulate the immune response differently than influenza A HA does [22,23]. After DNA electroporation, the IgG subclass distribution for the antibody responses to Env and HA revealed an excessive induction of IgG1 responses only for the Env antigen. The vaccine-induced polarization of T helper cells also differed between Env and HA [22]. In a model of allergic asthma, McAlees et al showed that CD4+ T cell subsets respond differentially to PD-1/PD-L1 blockade: overall regulation of CD4+ T cell responses by CPIs were complicated and strongly varied from strength of the TCR signaling and the initial T_H_1/T_H_2 status of the cell [51].

Surprisingly, although NP was a part of our influenza DNA-vaccine, in contrast to HA we observed no substantial CPI-mediated NP-specific IgG subtype modulation (Figure 3). HA and NP differ in a number of aspects, including their subcellular localization in the DNA transfected cells. This indicates that CPI-based genetic adjuvants have to be proven for each antigen/adjuvant DNA combination. By the co-application of CPIs with DNA vaccines encoding different viral transmembrane proteins, we observed comparable modulatory effects on the vaccine-induced IgG subclass distribution: from a T_H_2 [20] or T_H_1 (Figure 4) biased IgG1/IgG2a ratio towards a more balanced IgG subtype pattern.

Isotype class-switch in the antigen-experienced B cells as well as their following differentiation into plasma cells secreting high-affinity antibodies or memory B cells is supported and tightly regulated in the germinal centers (GC) by antigen-experienced CD4+ T follicular helper (TFH) cells [16]. It is known that localization and functionality of GC cells as well as the underlying T and B cell interactions during the germinal center response are controlled by the inhibitory receptor programmed cell death protein-1 and its ligands PD-L1 and PD-L2 [52,53]. To date, there is no clear concept of a vaccine-induced and adjuvant-mediated T_FH_ cell modulation strategy. In the context of HIV-1 immunization, Bradley et al. observed a modulatory capacity of immune checkpoint blockade on germinal center B and T follicular helper cells in macaques and mice [54]. Thus, immune checkpoint blockade during vaccine-induced interaction between follicular T and B cells might resemble a new strategy of adjuvant-mediated T_FH_ cell modulation.

Given the observed changes in HA-specific immune responses after checkpoint blockade, cancer patients treated with CPIs might react differently towards influenza vaccination. Läubli et al. showed, that influenza vaccination of lung cancer patients did not result in an altered vaccination efficacy compared to healthy individuals, but enhanced the frequency of immune-related adverse events (irAEs) [55]. In a more recent study with a higher number of enrolled patients, Keam et al. observed a significant increase of seroprotection against a variety of influenza strains in patients receiving checkpoint inhibitor therapy compared to patients receiving cytotoxic chemotherapy. Despite being less frequent, irAEs has been observed in patients receiving checkpoint inhibitor therapy [56]. Since cancer patients resemble a high-risk group for influenza complications, the safety of influenza vaccination during checkpoint inhibitor therapy needs to be further elucidated.

## 5. Conclusions

In the present study, we demonstrated that immune responses after IAV DNA immunization can be modulated by genetic checkpoint adjuvants. However, the modulation pattern and the modulatory CPI adjuvant differed from the DNA immunization against HIV-1 that was reported previously. Additionally, differences in the modulation of the immune responses against the two antigens (HA and NP) of the influenza DNA vaccine were observed. All this might indicate that CPI application serves as a fine-tuning tool for the vaccine-induced immune responses and is strongly dependent on the vaccine antigen and selected CPI adjuvant combination. Therefore, the modulatory effects of the immune checkpoint inhibition during vaccination against different IAV strains should be further validated in other animal models. Moreover, the effects of CPI treatment should be taken into consideration by seasonal IAV vaccination of cancer patients under CPI therapy.

## Supplementary Materials

The following are available online at https://www.mdpi.com/2076-393X/8/4/570/s1, Figure S1: CD8 T cell response. Percentage of CD8+ T cells producing IFNγ after in vitro stimulation with influenza HA and NP immunodominant MHC class I restricted peptides (measured by intracellular cytokine staining). Shown are mean values with SEM (*n* = 4), Figure S2: Generation and expression of soluble HA. (**A**) HA without transmembrane domain (TM) was cleaved out by enzymatic restriction and inserted into a pVax vector following a Kozak sequence and TPA leader sequence to improve secretion. (**B**) HA plasmids with and without TM were transfected in 293T cells. Three days after transfection, supernatants (SN) were collected, cells lysed and proteins analyzed by SDS-PAGE following sera staining and detection via an HRP-conjugated anti-mouse IgG antibody. (**C**) Soluble HA (coating antigen) produced in 293F cells with quality and purity validated by Western Blot and Silver staining, Figure S3: Quantitative amounts of HA-specific IgG1. HA-specific IgG1 amounts assessed by quantitative ELISA using a monoclonal HA IgG1 antibody as standard. Measured amounts of HA-specific IgG1 in ng/mL in sera of BALB/c mice after i.m. electroporation over a time-period of 18 weeks. Shown are mean values with SEM (*n* = 12–18) and significant differences between immunized groups (two-way ANOVA analyses followed by Tukey’s multiple comparison test, * *p* < 0.05 for sPD-L1 group compared to mock, ** *p* < 0.01 for sPD-1 group compared to mock, Figure S4: NP- and HA-specific IgG2b and IgG3 responses. NP-specific IgG2b (**A**) and IgG3 (**B**) and HA-specific IgG2b (**C**) and IgG3 (**D**) antibody responses in the sera of BALB/c mice after i.m. electroporation over a time-period of 18 weeks analyzed by ELISA. Shown are mean values with SEM (*n* = 9–18) and significant differences between the groups (two-way ANOVA analyses followed by Tukey’s multiple comparison test, (**D**) ** *p* < 0.01 for sPD-L1 group compared to sPD-1 group), Table S1: Vaccine-induced reciprocal neutralization titers against influenza A/PR/8/34 *.
