# Supplementary material for: Genetic Co-Administration of Soluble PD-1 Ectodomains Modifies Immune Responses against Influenza A Virus Induced by DNA Vaccination
Source: Vaccines (Basel). 2020 Oct 1;8(4):570. doi: 10.3390/vaccines8040570 (PMC7712647; doi:10.3390/vaccines8040570)
Supplement: Supplementary file 1 [file vaccines-08-00570-s001.pdf]

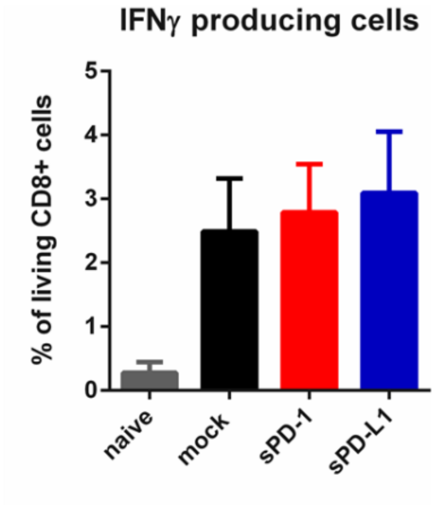

Figure S1. CD8 T cell response**.** Percentage of CD8+ T cells producing IFNγ after in vitro stimulation with influenza HA and NP immunodominant MHC class I restricted peptides (measured by intracellular cytokine staining). Shown are mean values with SEM (n = 4).

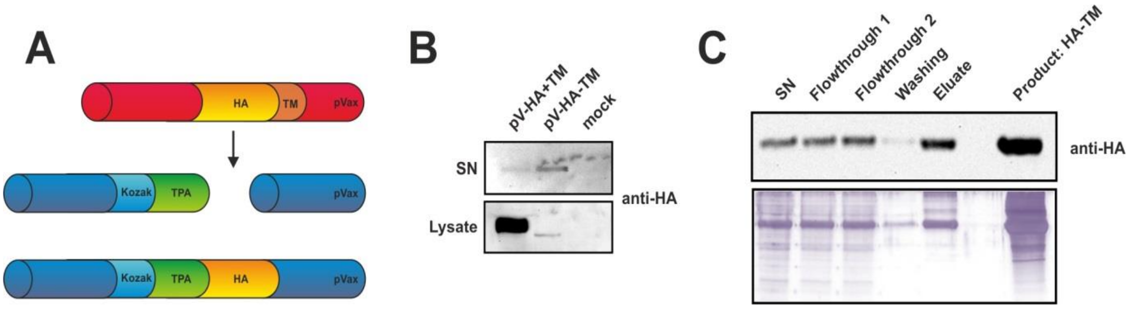

Figure S2. Generation and expression of soluble HA. (**A**) HA without transmembrane domain (TM) was cleaved out by enzymatic restriction and inserted into a pVax vector following a Kozak sequence and TPA leader sequence to improve secretion. (**B**) HA plasmids with and without TM were transfected in 293T cells. Three days after transfection, supernatants (SN) were collected, cells lysed and proteins analyzed by SDS-PAGE following sera staining and detection via an HRP-conjugated anti-mouse IgG antibody. (**C**) Soluble HA (coating antigen) produced in 293F cells with quality and purity validated by Western Blot and Silver staining.

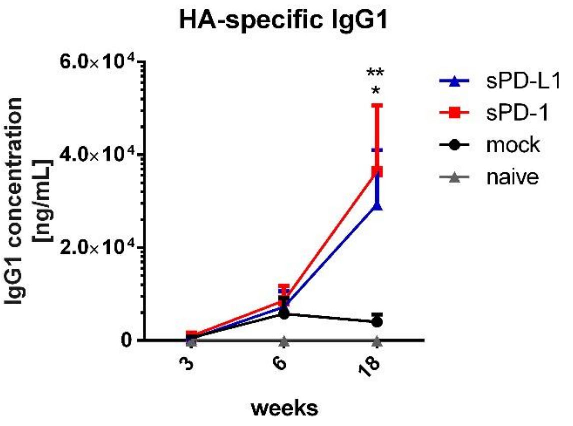

Figure S3. Quantitative amounts of HA-specific IgG1. HA-specific IgG1 amounts assessed by quantitative ELISA using a monoclonal HA IgG1 antibody as standard. Measured amounts of HA-specific IgG1 in ng/mL in sera of BALB/c mice after i.m. electroporation over a time-period of 18 weeks. Shown are mean values with SEM (n = 12–18) and significant differences between immunized groups (two-way ANOVA analyses followed by Tukey's multiple comparison test,* $p < 0.05$ for sPD-L1 group compared to mock, ** $p < 0.01$ for sPD-1 group compared to mock.

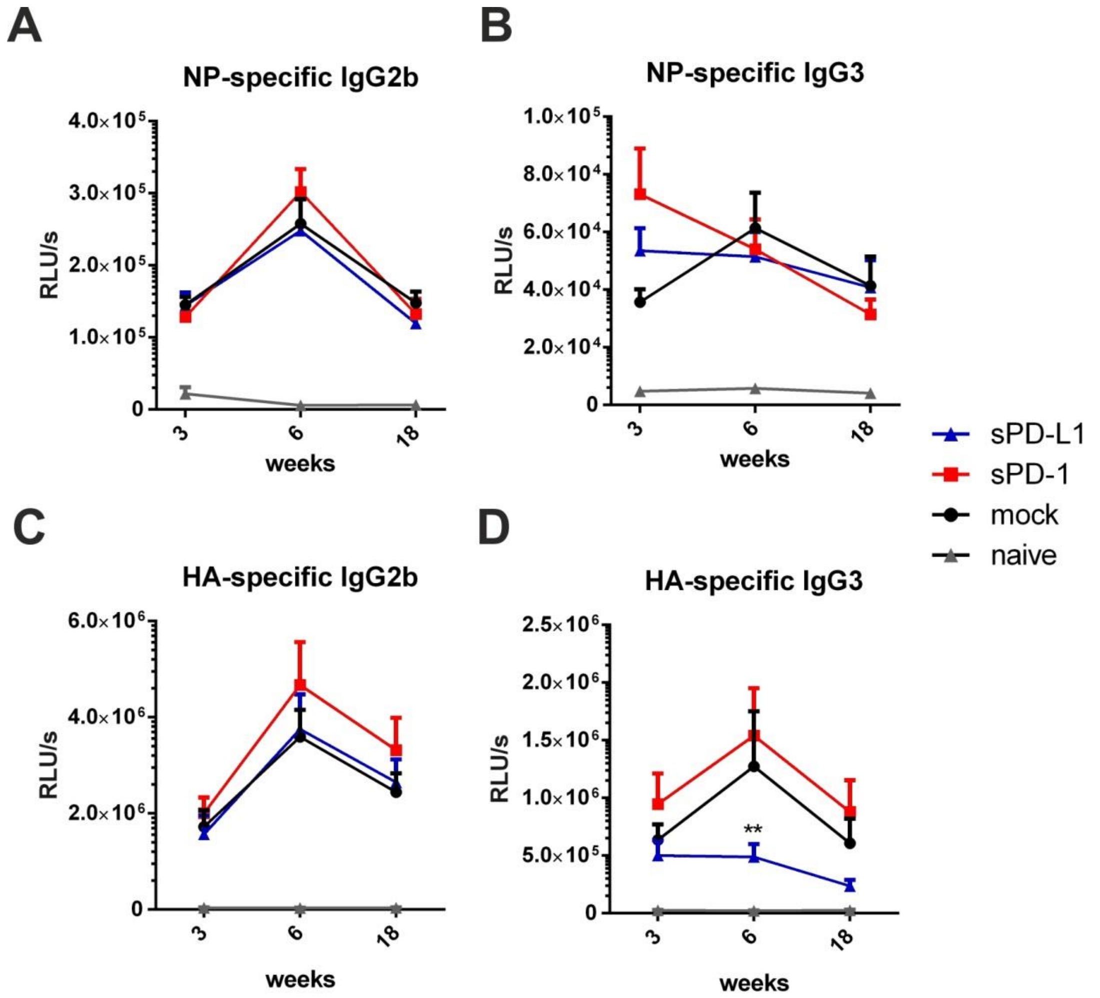

Figure S4. NP- and HA-specific IgG2b and IgG3 responses**.** NP-specific IgG2b (**A**) and IgG3 (**B**) and HA-specific IgG2b (**C**) and IgG3 (**D**) antibody responses in the sera of BALB/c mice after i.m. electroporation over a time-period of 18 weeks analyzed by ELISA. Shown are mean values with SEM (n = 9–18) and significant differences between the groups (two-way ANOVA analyses followed by Tukey's multiple comparison test, (**D**) ** $p < 0.01$ for sPD-L1 group compared to sPD-1 group).

Table S1. Vaccine-induced reciprocal neutralization titers against influenza A/PR/8/34 *.

	EXPERIMENTAL GROUPS			
**TITER**	**Naive**	**Mock**	**sPD-1**	**sPD-L1**
20	18	0	0	0
80	0	1	0	0
320	0	0	2	2
640	0	6	2	7
1280	0	9	7	5
2560	0	2	7	2
5120	0	0	0	1

* shown for serum samples obtained at week 18. Represents number of samples with corresponding neutralization titer for each experimental group.
